# Bone Mineral Metabolism Status, Quality of Life, and Muscle Strength in Older People

**DOI:** 10.3390/nu11112748

**Published:** 2019-11-12

**Authors:** Zoraida Verde, Andrea Giaquinta, Carmelo Moreno Sainz, Marta Díaz Ondina, Ana Fernández Araque

**Affiliations:** 1 Department of Biochemistry, Molecular Biology and Physiology, Universidad de Valladolid, Campus Duques de Soria, 42002 Soria, Spain; 2 Department of Nursery, Universidad de Valladolid, Campus Duques de Soria, 42002 Soria, Spain; agaranda1993@hotmail.com (A.G.); afa@enf.uva.es (A.F.A.); 3 Department of Clinic Biochemistry, Hospital Santa Bárbara, 42002 Soria, Spain; cmorenos@saludcastillayleon.es (C.M.S.); m.ondina@hotmail.com (M.D.O.)

**Keywords:** elderly, 25 hydroxy vitamin D, albumin, calcium, phosphorus, intact parathyroid hormone, EuroQoL-5 dimensions, physical activity, Mini Nutritional Assessment

## Abstract

As the relationship between vitamin D and various diseases or health conditions has become known, interest in the contribution of vitamin D to overall health-related quality of life (QoL) has increased. We examined the relationship between vitamin D status and QoL in 273 participants aged 65 years and older. Serum levels of total calcium, phosphorus, intact parathyroid hormone, albumin, and 25-hydroxyvitaminD3 were analyzed. We also recruited data for QoL, physical activity, nutritional impairment, and muscular strength. Ninety percent of the subjects were classified as vitamin D deficient or insufficient. Participants with higher serum 25(OH)D3, calcium, phosphorous, and Alb levels were significantly less likely to self-report depression or anxiety after adjustment (*p* = 0.009, *p* = 0.005, *p* = 0.003, and *p* = 0.005, respectively). Additionally, we found an association between lower levels of albumin and self-reported problems with mobility or usual activities (*p* = 0.01). We also found associations between better muscle strength and higher levels of vitamin D, calcium, phosphorous, and albumin (*p* = 0.006, *p* = 0.003, *p* = 0.004 and *p* = 0.002, respectively). Overall, our data provide evidence that serum vitamin D and Alb levels are negatively related to self-reported anxiety or depression, usual activities, mobility, and three dimensions of QoL in older adults. Furthermore, vitamin D levels are positively related to hand grip strength in adults over 65 years old.

## 1. Introduction

Vitamin D deficiency is a major public health problem worldwide, in all age groups, with particular emphasis in at risk groups. Vitamin D is involved in the absorption and distribution of calcium (Ca^2+^), and correlations between vitamin D and bone health and Ca^2+^ homeostasis have been proven by numerous studies [1,2,3]. In addition, more extraskeletal effects of vitamin D have been revealed, and the diverse functions of vitamin D have also been supported by the discovery that vitamin D receptors (VDRs) and vitamin D activating enzymes (hydroxylases) are present in the tissues and cells not involved in mineral and bone metabolism. Because VDRs have been located in multiple tissues, vitamin D deficiency has been associated with an increased risk of chronic diseases such as cardiovascular disease, diabetes, and dementia [4,5,6,7].

The essential role of Vitamin D for older adults is well known. Ca^2+^ homeostasis is altered in the majority of older adults, especially with chronic kidney disease (CKD), and is manifested predominantly as hypocalcemia, hyperphosphatemia, vitamin D deficiency, fibroblast growth factor-23 (FGF23) elevation (coupled with Klotho deficiency), and secondary hyperparathyroidism. These defects are deleterious to bone and soft-tissue health and lead to the development of metabolic bone disorders, which are associated with aging and morbid clinical outcomes, including fracture, cardiovascular events, and mortality [5,8,9,10]. Hence, recent studies have suggested a relationship between vitamin D and measurements of health conditions. In the last decade, improvements in health systems have resulted in populations living longer [11], however, aging is a complicated process that may result in the loss of functional health, muscle weakness, and disability due to variations in skeletal muscle quantity and quality [12]. Muscle strength is a useful index of declining mobility and disability which are included in the definition of frailty status and are associated with quality of life (QoL) in several populations [13].

In this context, vitamin D has an important role for older adults who are at high risk of deficiency, and therefore adequate intakes of vitamin D should be ensured as a matter of public health. This study aimed, first, to examine the values of vitamin D in a representative sample of the elderly Spanish population (over 65 years) and, secondly, to examine the relationship between serum bone mineral biomarkers and QoL or physical function status.

## 2. Methods

### 2.1. Study Design

This study was part of a cross-sectional study carried out from January 2018 to May 2018 and from January 2019 to May 2019 in a representative cohort of individuals aged 65 and older in the north of Spain (Soria). The study was conducted according to the guidelines laid down in the Declaration of Helsinki and approved by the Area de Salud de Burgos y Soria Ethics Committee (Ref. CEIC 1446). Written informed consent was obtained from all subjects and signed prior to testing. The inclusion criteria were adults over 65 years old, not institutionalized, and free of renal illness, dementia, mobility impairments, or chronic disorders that could affect bone mineral metabolism. The Mini-Mental State Examination (MMSE) was used to screen possible cognitive issues and mobility was evaluated based on their ability to walk, without any aid, more than one minute. For the estimating sample size, we considered type one (α) and type two errors (β) of 0.05 and 0.20 (power = 80%), d = 5. We calculated a sample size of 235 participants, based on previous data of vitamin D deficiency prevalence (plasma 25-hydroxyvitamin D3 (25-OH-D3) < 20 ng/mL) in older adults.

### 2.2. Procedures

Two hundred and seventy-five older adults (over 65 years old) were recruited for the study. Selected participants who were being treated at different primary care centers were interviewed by a research nurse and the following data were collected: demographics, anthropometrics, cigarette smoking, drugs prescribed, falls and hospital admissions during the last year, and clinical group risk (CGR) category. The CRGs category is a claims-based classification system for risk adjustment that assigns each individual to a single mutually exclusive risk group based on historical clinical and demographic characteristics to predict future use of healthcare resources.

#### 2.2.1. Questionnaires

The quality of life measures included the EuroQoL-5 dimensions (EQ-5D) which is a standardized measure of health-related quality of life (QoL) that can be used in a wide range of health conditions and treatments. This descriptive system is comprised of the following five dimensions: mobility, self-care, usual activities, pain or discomfort, and anxiety or depression. The EQ-5D-visual analogue scale (EQ-VAS) records an individual’s self-rated health on a vertical visual analogue scale. This was used as a quantitative measure of health outcome that reflected the subjects’ own judgement. The EQ-VAS records the respondent’s self-rated health on a 20 cm vertical, visual analogue scale with endpoints labelled “the best health you can imagine” and ”the worst health you can imagine” [14].

The physical activity questionnaire used was the Physical Activity Scale for the Elderly (PASE), which is a brief (5 min) and easily scored survey designed specifically to assess physical activity in epidemiological studies of adults aged 65 years and older. The PASE score combines information on leisure, household, and occupational activity, as well as assesses the types of activities typically chosen by older adults (walking, recreational activities, exercise, housework, yard work, and caring for others). It uses frequency, duration, and intensity level of activity over the previous week to assign a score, ranging from 0 to 793, with higher scores indicating greater physical activity [15].

To assess nutritional status, the Mini Nutritional Assessment (MNA) is able to classify older adults as well nourished, at risk for malnutrition, or malnourished. The MNA consists of 18 self-reported questions derived from the following four parameters of assessment: anthropometric assessment, general assessment, dietary assessment, and self-assessment. We performed the full MNA for all subjects [16].

#### 2.2.2. Physical Performance Measures

Muscular strength was assessed using the hand grip strength test. After adjustment for hand size, three measures were performed with the dominant hand and were averaged for the analysis [17]. Analyses of grip strength were undertaken by age and gender. The European Working Group on Sarcopenia in Older Persons defined weakness based on a grip strength less than 30 kg in men and less than 20 kg in women [18]. For the identification of participants with clinically meaningful weakness, handgrip strength was classified in two categories as follows: weak-intermediate and normal, according to cut-off values published by Alley et al., 2014 [19].

#### 2.2.3. Biochemical Blood Analysis

Blood samples were obtained by venipuncture, in the morning, by a trained nurse. Blood samples were sent to the Hospital Santa Bárbara Biochemistry Service. Bone mineral metabolism biomarkers (serum total Ca^2+^, phosphorus, intact parathyroid hormone (iPTH), albumin (Alb), creatinine, and 25-OH-D3 levels) were analyzed. Serum total Ca^2+^, Alb, and creatinine levels were analyzed by molecular absorption spectrometry on a Cobas 8000 c702 analyzer by Roche (Basel, Switzerland). The iPTH, phosphorus, and 25-OH-D3 levels were determined using electro chemiluminescent immunoassay (ECLIA) using Cobas e411 and Cobas 8000 e602 analyzers by Roche (Basel, Switzerland) respectively.

### 2.3. Statistical Analyses

Demographic and clinical data were described as mean and standard deviation (SD) for continuous variables and frequencies (percentages) for categorical data. Independent sample *t*-tests were used for continuous variables and Chi-square tests for categorical variables. The correlation between variables was measured by calculating the linear coefficient of correlation (Pearson’s r) and the regression by designing multiple linear regression models. Associations between physical performance measures (independent variables) with dependent variables were analyzed with binary logistic regression.

All statistical analyses were corrected for multiple comparisons using the Bonferroni method, in which the threshold *p*-value is obtained by dividing 0.05 by the number of tests. Data were analyzed using the PASW/SPSS Statistics 24.0 (SPSS Inc, Chicago, IL, USA) program.

## 3. Results

Of the 275 adults assessed for eligibility, two adults presented with creatinine greater than 1.9 mg/dL, Ca^2+^ greater than 10.9 mg/dL, or phosphorus less than 2.5 mg/dL, and therefore were excluded. Of the subjects recruited, 47.1% were male. The mean age was 75.74 ± 7.16 years (with a range of 65–94 years). Thirty-three per cent of the participants were in the normal range for body mass index (BMI), 42.5% were in the overweight range, and 24.5% were obese. On the basis of the CGR classification, 68.3% of the participants presented a good health status or no chronic diseases or any pluripathology, 73.4% were non-smokers, 21.0% were ex-smokers, and 5.6% were smokers. The mean for drugs consumed was 3.99 ± 2.93 and 37.4% presented polypharmacy, i.e., 37.4% taking more than five medications. In addition, we observed that 14.7% of the subjects had vitamin D supplementation. The EQ-5D and EQ-VAS means were 0.81 ± 0.16 and 71.45 ± 16.07, respectively.

The mean serum 25(OH) D3 level was 18.48 ± 8.87 ng/mL (with a range of 3.00–68.46 ng/mL). According to the Institute of Medicine (IOM) cut-off points, 64.8% of the subjects enrolled were classified as vitamin D deficient (≤20 ng/mL), 25.1% as insufficient (range 21–29 ng/mL), and 10.1% as adequate (≥30 ng/mL).

Table 1 shows the baseline and clinical characteristics of the participants divided by gender. We observed significant differences between gender and falls, dimension 5 of EQ-5D (problems with depression or anxiety), EQ-VAS, and hand grip measure, (*p* < 0.001, *p* = 0.009, *p* = 0.002, and *p* < 0.001, respectively). In addition, females presented significantly higher levels of Ca^2+^ and phosphorus in plasma as compared with males.

Table 2 presents the results of the Pearson’s correlation between crude variables. As expected, we observed a negative correlation between age and hand grip test, EQ-5D, creatinine, albumin or PTH (*p* < 0.001, *p* = 0.009, *p* < 0.001, and *p* < 0.001, respectively). In addition, we observed a marginal negative correlation between age and EQ-VAS, MNA, and vitamin D (*p* = 0.023, *p* = 0.023, and *p* = 0.019, respectively).

For the number of drugs consumed, we observed a negative correlation with PASE, EQ-5D, and EQ-VAS (*p* = 0.003, *p* < 0.001, and *p* < 0.001, respectively). In addition, we found a positive correlation between physical activity levels and EQ-VAS (*p* < 0.001) and a negative correlation with the nutritional status (MNA) (*p* < 0.001). We did not find any correlations between PASE and biochemical parameters.

**Figure 1:**
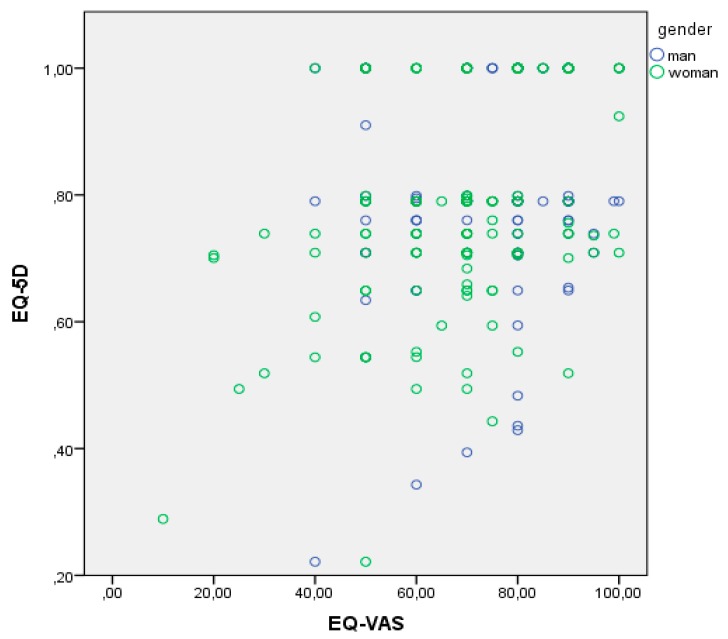
Scatter plot of the correlation between EQ-5D and EQ-VAS divided by gender. Abbreviations: EQ-5D, EuroQoL-5 dimensions; EQ-VAS, EQ-5D-visual analogue scale.

It is important to highlight that there was a significant correlation between EQ-5D and EQ-VAS, there was agreement between subjects’ description of personal well-being, represented by the extent of health problems in five dimensions and what the subjects thought about the health state that they had self-reported (Figure 1).

As expected, we observed a negative correlation between MNA and hand grip test (p < 0.001) (Table 2). In the case of QoL and biomarkers, we only observed a marginal negative correlation between EQ-VAS and phosphorus levels (*p* = 0.008) (Table 2). For muscle strength and biomarkers, we observed a negative correlation between hand grip values and phosphorus and a positive correlation with Alb levels (*p* < 0.001 and *p* < 0.001, respectively) (Table 2).

### Associations between EQ-5D, Hand Grip, and MNA and Mineral Metabolism Biomarkers

Table 3 shows the adjusted associations between biomarker levels and having problems in each of the five EQ-5D dimensions, hand grip strength, and nutritional status. For the EQ-5D, we observed significant associations between levels of Ca^2+^, phosphorous, and Alb and self-report of any problem, as well as a marginal association between lower levels of 25(OH) D3 and self-report of any problem.

On the one hand, attending each dimension of EQ-5D, we found statistically significant associations between lower levels of 25(OH) D3, Ca^2+^, phosphorous, and Alb and self-reported anxiety or depression. In addition, we found a marginal significant association between lower levels of vitamin D or Alb and self-reported problems with mobility or usual activities. We also found associations between better muscle strength and higher levels of 25(OH) D3, Ca^2+^, phosphorous, and Alb. On the other hand, we did not find any associations between EQ-VAS or MNA and the biomarkers analyzed.

## 4. Discussion

In this study, we found a high prevalence (89.9%) of deficient or insufficient serum levels of vitamin D (serum 25(OH)D3, <30 ng/mL) in a group of 273 elderly subjects, representative of a healthy age-related Spanish population. Our results are similar to previous studies in Spanish populations [19,20,21,22].

This is the first study to analyze associations of a more complete selection of bone mineral metabolism biomarkers in an older population (>65 years) with each of the five dimensions of quality of life (EQ-5D). The older population is defined as adults aged 65 years and over, and therefore it is important to select and focus on a specific vitamin D sensitive population instead of a group that is not well defined. Furthermore, following international guideline recommendations such as the International Federation of Clinical Chemistry and Laboratory Medicine, for the analysis of vitamin D levels, it is mandatory to measure other bone mineral metabolism biomarkers or creatinine levels in order to verify results and exclude possible pathologies that could have modified vitamin D levels [23].

The EQ-5D is a standardized method used to evaluate health status in a wide range of health conditions and treatments. Dimension 5 reflects the impact of common mental health conditions, such as mild to moderate depression or anxiety, on daily function and it has been found to be sensitive to detect improvements in treated patients with depression [24].

The EQ-5D results were similar to published studies in Spanish populations [25]. Dividing by gender, the percentage of individuals with problems in each dimension of the EQ-5D and the dimension of mobility presented a similar distribution; ≥26.6% of individuals with problems. For self-care, usual activities, and pain dimensions, there was a higher percentage of individuals with problems in the female group. The dimension of anxiety or depression was the one that showed the highest differences according to gender. We also observed a relationship between EQ-5D and age. Gender and age are established determinants of vitamin D status and quality of life. Therefore, it is important to include the corrections in any analysis [25,26,27].

On the basis of the EQ-5D results, we observed that bone mineral metabolism biomarkers were associated with three of five dimensions of EQ-5D, as well as with self-reported QoL. The absence of a statistically significant association with self-care or a marginal association with usual activities could be due to the low prevalence of reported problems with these items in the studied population. Confounding by indication is a frequently encountered bias in observational epidemiologic studies, which can led to an underestimation of problems with pain and discomfort and explains why a marginal association for this dimension was observed [28].

Our study revealed significant associations of lower 25(OH) D3, Ca^2+^, phosphorus, and Alb levels and self-reported problems with depression or anxiety. Nutrition and dietary habits have been related to the occurrence of depression or anxiety [29,30,31]. Several authors have found that vitamin D deficiency was highly prevalent among depressed or anxious patients, and supplementation had a significant influence on mental health improvements, particularly for depression [25,26].

From a biological point of view, numerous recent studies have identified VDRs in nearly all tissues in the body, including both neuronal and glial cells in the central nervous system [32]. In addition, the enzymes necessary for the hydroxylation of 25(OH)D3 to the active form are present in the hypothalamus, cerebellum, and substantia nigra [33]. Vitamin D modulates the hypothalamic-pituitary-adrenal axis, regulating adrenalin, noradrenaline, and dopamine production through VDRs in the adrenal cortex and protects against the depletion of dopamine and serotonin centrally [34,35]. Therefore, biological plausibility for the action of vitamin D in depression has been established.

Epidemiological surveys show that approximately 8% and 3% of adults over 65 years of age still meet formal diagnostic criteria for depression or anxiety disorders, respectively [31,32,33]. This is significant given that the number of adults over 65 years is projected to increase in the coming decades and given these conditions compound the effects of physical comorbidities [36,37,38,39]. In addition, it should be noted that Simon Spedding et al. demonstrated, in a meta-analysis study, that the effect size for vitamin D in depression is comparable with the effect of antidepressant medication, an accepted treatment for depression [40].

Regarding the EQ-5D dimensions, in our study, we also observed an association between self-reported problems with mobility or usual activities and lower levels of serum Alb. Recently, Uemura et al. reported that sarcopenia and low serum Alb level independently and synergistically increase the risk of incident disability [41]. In addition, previous studies have suggested that a low serum Alb level is related to reduced muscle mass and muscle strength in older adults [37,38]. Therefore, muscle mass or function (strength and performance) may decline as a result of degradation of protein synthesis caused by malnutrition, related to albumin levels [41]. Despite this relationship, we did not find any association between nutritional status and Alb levels. This is probably because most of the subjects of our study (89.0%) had an adequate nutritional status (MNA > 24), only 11.1% were at risk of malnutrition (MNA between 17 and 23.5), and no one suffered from protein-calorie malnutrition.

Hand grip strength has been used in studies as an indicator of general muscle strength and frailty [42,43,44]. The identification of factors associated with the reduction in muscle strength and physical frailty among elderly adults provides important resources to plan health care for this population, which is highly heterogeneous in terms of prevalence of physical frailty conditions.

In our study lower levels of vitamin D, Ca^2+^, phosphorus, and Alb were significantly associated with the decline of muscle strength, which was measured using hand grip strength.

Vitamin D is a fat-soluble vitamin which is crucial for muscle and bone function, among many other physiological roles. Low serum vitamin D is linked to reduced physical functioning and frailty development, as well as falls and mortality. It is well known that a deficiency of vitamin D has resulted in muscle weakness and a significant reduction in muscle force has been noted when vitamin D deficiency was accompanied by reduced levels of Ca^2+^ [45]. Vitamin D stimulates the absorption of Ca^2+^ from the intestine and maintains the serum Ca^2+^ levels that are required for normal bone mineralization and for the maintenance of muscle function [46].

Previous studies have revealed controversial results regarding the relationship between vitamin D and hand grip strength [4,47,48]. Our results could contribute to clarify the association between vitamin D and hand grip strength, providing more complete information about bone metabolism biomarkers in an older population which was not considered in other studies [4,42,43,44]. In addition, our results were adjusted for a set of covariables, such as age and gender.

Overall, vitamin D is the principal factor that maintains Ca^2+^ homeostasis. Increasing evidence indicates that the reason for a disturbed Ca^2+^ balance with age is inadequate vitamin D levels in the elderly. Vitamin D stimulates intestinal absorption and kidney reabsorption of Ca^2+^ and phosphorus, mainly through its dominant active metabolite 1, 25-dihydroxyvitamin D3 (1,25(OH)2D3). In the parathyroid gland, vitamin D suppresses PTH production. Consequently, low circulating vitamin D levels invariably result in elevated serum PTH concentrations and poorer reabsorption of Ca^2+^ and phosphorus in healthy individuals [49,50]. It should be mentioned that, in the general population, vitamin D status has been defined by either a single criterion or a combination of criteria with changes in several of them such as: serum PTH concentration, circulating 25(OH)D3 levels, intestinal Ca^2+^ and phosphorus absorption, muscle strength, and bone mineral density [51,52]. Hence, we think it is mandatory to obtain complete information of bone mineral biomarkers.

On the one hand, this study has several strengths. First, this study evaluates the influence of a complete background of bone mineral serum biomarkers on quality of life and muscle strength in a population of older adults (over 65 years old). Secondly, we have controlled a wide set of important confounders such as demographic variables, nutritional status, lifestyle factors, CGR classification, and season of serum 25(OH) D3 measurement. Thirdly, we have measured MMSE status and creatinine, Ca^2+^, or phosphorus levels in order to exclude possible pathologies that could have modified vitamin D levels. Fourthly, there are no studies that recover both scores, EQ-5D and EQ-VAS, which complete objective self-reported data with subjective scores, checking a correct phenotype of the participants. On the other hand, this study has several limitations. There were 14.7% of the subjects receiving vitamin D supplementation, we had a controlled environment (season) and other contributing factors but we did not recover (type, dose, adherence and duration of vitamin D supplementation), however, we did not observe any significant differences in levels of vitamin D in both groups. We only selected 273 noninstitutionalized elderly subjects (65–94 years) from the North of Spain, however, we believe this can be partly overcome by the fact that our population is homogeneous, not stratified, and well defined in terms of phenotype assessment. Thus, further cohorts of different ages and experimental studies are needed. Another possible limitation was the use of self-reported information, however, we used validated questionnaires and a research nurse helped participants, keeping it short and easy to complete.

## 5. Conclusions

Our data provide evidence that serum vitamin D levels are related to older adults self-reported QoL. In addition, on the one hand, vitamin D and Alb levels are negatively related to self-reported problems with anxiety or depression, usual activities or mobility, and three dimensions of QoL in elderly adults. On the other hand, 25(OH)D3 is positively related to hand grip strength in adults over 65 years old. This study suggests that older adults with inadequate levels of vitamin D should be identified early, and therefore future functional decline, problems with QoL, and several adverse health events could be prevented. Vitamin D intake could be a strategy to attenuate age-dependent poor health outcomes. Further studies are required to overcome these strategies and strengthen its benefits for QoL among older adults.

## Figures and Tables

**Table 1:** Characteristics of the study population.

Characteristics	Men *n* = 129	Women *n* = 144	*p*-Value
Age, mean (SD)	76.14 (7.32)	75.40 (7.04)	0.394
BMI (SD)	27.62 (3.77)	27.80 (4.23)	0.728
Total drugs used, mean (SD)	3.88 (2.83)	4.11 (3.01)	0.522
Falls (SD)	0.07 (0.26)	0.26 (0.52)	**<0.001**
Hospital admissions (SD)	0.10 (0.29)	0.09 (0.28)	0.770
CRG (% pluripathologic or chronic diseases)	34.10	29.90	0.268
EQ-5D (SD)	0.83 (0.16)	0.79 (0.16)	0.058
EQ-5D, problems with mobility (%)	26.6	31.3	0.237
EQ-5D, problems with self-care (%)	3.1	9.7	0.024
EQ-5D, problems with usual activities (%)	7.8	14.6	0.058
EQ-5D, problems with pain and discomfort (%)	39.8	51.4	0.037
EQ-5D, problems with depression or anxiety (%)	25.0	39.6	**0.009**
EQ-5D, any problem (%)	61.7	70.8	0.072
EQ-VAS, mean (SD)	74.64 (14.03)	68.63 (17.30)	**0.002**
VAS, mean (SD)	2.51 (2.53)	2.97 (2.51)	0.131
PASE (SD)	329.68 (199.82)	331.13 (196.59)	0.646
Hand grip, mean (SD)	45.21 (26.23)	30.47 (16.22)	**<0.001**
MNA, mean (SD)	27.06 (2.60)	26.74 (2.97)	0.350
Vitamin D, mean (SD)	19.83 (7.43)	20.19 (3.00)	0.796
PTH (SD)	64.32 (27.47)	67.51 (29.77)	0.369
Ca^2+^ (SD)	9.40 (0.30)	9.52 (0.37)	**0.007**
Phosphorus (SD)	3.06 (0.43)	3.42 (0.49)	**<0.001**
Creatinine (SD)	1.04 (0.23)	0.82 (0.19)	**<0.001**
Albumin (SD)	4.45 (0.25)	4.39 (0.25)	0.044

Note: Values are percentages for categorical data or mean and standard deviation for continuous data. Statistically significant variables are in bold. Abbreviations: SD, standard deviation; BMI, body mass index; CRG, clinical risk groups; EQ-5D, EuroQoL-5 dimensions; EQ-VAS, EQ-5D-visual analogue scale; VAS, visual analogue scale; PASE, Physical Activity Scale for the Elderly; MNA, Mini Nutritional Assessment; PTH, parathyroid hormone; Ca^2+^, calcium.

**Table 2:** Pearson correlation coefficients of analyzed variables.

Variables	Age	BMI	Number of Drugs	Number of Falls	Number of Hospitalizations	PASE	Hand Grip	EQ-5D	EQ-VAS	MNA	Vitamin D	Ca2+	phosphorus	Alb	iPTH
**Age**	1	−0.047	−0.006	0.016	0.016	0.003	−0.255	−0.159	−0.137	−0.138	−0.143	−0.025	−0.092	−0.301	0.249
	−	0.444	0.920	0.792	0.792	0.967	**<0.001**	**0.009**	**0.023**	**0.023**	**0.019**	0.678	0.132	**<0.001**	**<0.001**
**BMI**		1	0.096	0.010	−0.041	−0.115	0.082	−0.108	−0.062	0.060	0.059	0.026	−0.026	0.063	−0.012
		−	0.115	0.871	0.507	**0.050**	0.178	0.076	0.310	0.327	0.338	0.672	0.677	0.302	0.845
**Number of Drugs**			1	0.020	0.032	−0.182	−0.116	−0.327	−0.356	−0.019	0.070	0.058	0.102	−0.027	0.020
			−	0.747	0.606	**0.003**	0.056	**<0.001**	<0.001	0.753	0.253	0.347	0.094	0.661	0.748
**Number of Falls**				1	0.157	−0.075	−0.081	−0.039	−0.109	−0.005	0.057	0.056	0.129	−0.049	0.051
				−	**0.011**	0.221	0.189	0.529	0.075	0.929	0.356	0.363	**0.036**	0.431	0.418
**Number of Hospitalizations**					1	0.054	0.072	−0.027	−0.027	−0.004	0.079	0.021	0.107	−0.005	−0.096
					−	0.378	0.242	0.666	0.657	0.945	0.205	0.733	0.086	0.936	0.123
**PASE**						1	−0.107	0.397	0.221	−0.225	−0.017	−0.044	−0.024	0.062	0.006
						−	0.078	**<0.001**	**<0.001**	**<0.001**	0.777	0.470	0.697	0.310	0.925
**Hand Grip**							1	0.107	0.119	0.371	0.001	−0.014	−0.217	0.212	0.138
							−	0.078	**0.050**	**<0.001**	1	0.817	**<0.001**	**<0.001**	**0.024**
**EQ-5D**								1	0.309	0.026	0.002	−0.072	−0.045	0.018	−0.007
								−	**<0.001**	0.672	0.975	0.237	0.467	0.769	0.910
**EQ-VAS**									1	0.096	0.006	0.009	−0.160	0.134	−0.046
									−	0.115	0.927	0.887	**0.008**	**0.028**	0.459
**MNA**										1	0.038	−0.108	−0.080	0.058	0.035
										−	0.537	0.077	0.191	0.347	0.567
**Vitamin D**											1	0.023	0.123	0.056	−0.196
											−	0.704	**0.044**	0.360	**0.001**
**Ca^2+^**												1	0.052	0.471	−0.035
												−	0.393	**<0.001**	0.575
**phosphorus**													1	−0.007	−0.090
													−	0.910	0.144
**Alb**														1	−0.209
														−	**0.001**
**iPTH**															1
															−

Note: Each cell contains two values: (a) Pearson correlation coefficient and (b) *p*-value of testing if the correlation is significant. Statistically significant variables are in bold. Abbreviations: BMI, body mass index; PASE, Physical Activity Scale for the Elderly; EQ-5D, EuroQoL-5 dimensions; EQ-VAS, EQ-5D-visual analogue scale; MNA, Mini Nutritional Assessment; Ca^2+^, calcium; Alb, albumin; iPTH, intact parathyroid hormone.

**Table 3:** Logistic regression of association between bone mineral metabolism biomarkers and each of the EQ-5D dimension problems, EQ-VAS, hand grip strength, and nutritional status.

Measurements	Vitamin D			Ca^2+^			phosphorus			Alb			iPTH		
	Adjusted OR	95% CI	*p*-Value	Adjusted OR	95% CI	*p*-Value	Adjusted OR	95% CI	*p*-Value	Adjusted OR	95% CI	*p*-Value	Adjusted OR	95% CI	*p*-Value
EQ-5D *															
Mobility	0.964	0.933–0.995	0.024	0.918	0.859–0.981	0.011	0.768	0.627–0.942	0.011	0.835	0.727–0.959	**0.010**	0.990	0.981–1.000	0.044
Self-care	0.943	0.875–1.016	0.125	0.805	0.739–1.013	0.072	0.647	0.398–1.051	0.078	0.734	0.526–1.025	0.069	0.985	0.964–1.006	0.158
Usual activities	0.924	0.866–0.987	0.019	0.845	0.743–0.962	0.011	0.598	0.401–0.893	0.012	0.698	0.530–0.917	**0.010**	0.979	0.960–0.997	0.025
Pain and discomfort	0.983	0.958–1.009	0.206	0.946	0.894–1.000	0.050	0.833	0.702–0.989	0.037	0.894	0.796–1.005	0.060	0.991	0.983–0.999	0.034
Depression or anxiety	0.959	0.930–0.990	**0.009**	0.912	0.856–0.972	**0.005**	0.739	0.606–0.901	**0.003**	0.825	0.722–0.943	**0.005**	0.988	0.979–0.998	0.014
Any problem	0.974	0.948–1.000	0.049	0.926	0.875–0.981	**0.009**	0.779	0.655–0.927	**0.005**	0.852	0.756–0.960	**0.008**	0.990	0.982–0.998	0.014
EQ-VAS	1.036	0.994–1.080	0.095	1.046	0.967–1.132	0.262	1.142	0.897–1.453	0.281	1.112	0.942–1.313	0.211	1.005	0.993–1.016	0.149
Hand grip	1.035	1.010–1.060	**0.006**	1.087	1.028–1.150	**0.003**	1.263	1.078–1.480	**0.004**	1.206	1.070–1.358	**0.002**	1.004	0.997–1.011	0.309
MNA	1.008	0.966–1.051	0.727	1.010	0.925–1.102	0.829	1.038	0.793–1.360	0.784	1.019	0.842–1.220	0.843	1.002	0.989–1.015	0.755

* Problems in each dimension of EQ-5D. Statistically significant variables are in bold. Abbreviations: EQ-5D, EuroQoL-5 dimensions; EQ-VAS, EQ-5D-visual analogue scale; MNA, Mini Nutritional Assessment; Ca^2+^, calcium; Alb, albumin; iPTH, intact parathyroid hormone.

## Abbreviations

Alb	Albumin
BMI	Body mass index
Ca^2+^	Calcium
CGR	Clinical group risk
CKD	Chronic kidney disease
1,25(OH)2D3	1, 25-dihydroxyvitamin D3
EQ-5D	EuroQoL-5 dimensions
EQ-VAS	EQ-5D-visual analogue scale
FGF23	Fibroblast growth factor-23
25 (OH)D3	25-hydroxyvitamin D3
IOM	Institute of Medicine
iPTH	Intact parathyroid hormone
MMSE	Mini Mental State Examination
MNA	Mini Nutritional Assessment
PASE	Physical Activity Scale for the Elderly
QoL	Quality of life
SD	Standard deviation
VDRs	Vitamin D receptors
